# COVID-19-induced acute kidney injury and chronic kidney disease: correspondence response

**DOI:** 10.1590/2175-8239-JBN-2022-0115rep-en

**Published:** 2022-12-16

**Authors:** Carlos G. Musso, Gustavo Aroca-Martinez

**Affiliations:** 1 Universidad Simón Bolivar, Facultad de Ciencias de la Salud, Barranquilla, Colombia.; 2 Hospital Italiano de Buenos Aires, Departamento de Investigación, Buenos Aires, Argentina.

Dear Editor,

Regarding the comment by Dr. Rujittika Mungmunpuntipantip and Dr. Viroj Wiwanitkit, we would like to point out that the initial classification of all the studied individuals into kidney-healthy and chronic kidney disease (CKD) patients was based on their electronic medical records (physical exams, laboratory tests, and abdominal images), as explained in the “Material & Method” section of our article. This was possible because these patients were usually treated in our medical institution. Therefore, all CKD diagnoses performed were based on the KDIGO criteria^1^.

Regarding COVID-19 infection, we focused on those patients who presented acute COVID-19 symptoms and a positive PCR test. The other COVID-19 conditions (asymptomatic, subclinical, etc.) were not of interest for our study.
